# Effects of powered ankle–foot orthoses mass distribution on lower limb muscle forces—a simulation study

**DOI:** 10.1007/s11517-023-02778-2

**Published:** 2023-01-23

**Authors:** Grace Marconi, Alpha Agape Gopalai, Sunita Chauhan

**Affiliations:** 1 Department of Mechanical and Aerospace Engineering, Monash University, Clayton, Australia; 2 School of Engineering, Monash University, Selangor, Malaysia

**Keywords:** Gait analysis, Biomechanics, Ankle–foot orthosis, Musculoskeletal modelling, Kinetics, Foot drop

## Abstract

This simulation study aimed to explore the effects of mass and mass distribution of powered ankle–foot orthoses, on net joint moments and individual muscle forces throughout the lower limb. Using OpenSim inverse kinematics, dynamics, and static optimization tools, the gait cycles of ten subjects were analyzed. The biomechanical models of these subjects were appended with ideal powered ankle–foot orthoses of different masses and actuator positions, as to determine the effect that these design factors had on the subject’s kinetics during normal walking. It was found that when the mass of the device was distributed more distally and posteriorly on the leg, both the net joint moments and overall lower limb muscle forces were more negatively impacted. However, individual muscle forces were found to have varying results which were attributed to the flow-on effect of the orthosis, the antagonistic pairing of muscles, and how the activity of individual muscles affect each other. It was found that mass and mass distribution of powered ankle–foot orthoses could be optimized as to more accurately mimic natural kinetics, reducing net joint moments and overall muscle forces of the lower limb, and must consider individual muscles as to reduce potentially detrimental muscle fatigue or muscular disuse.

**Graphical Abstract:**

OpenSim modelling method to explore the effect of mass and mass distribution on muscle forces and joint moments, showing potential mass positioning and the effects of these positions, mass, and actuation on the muscle force integral.

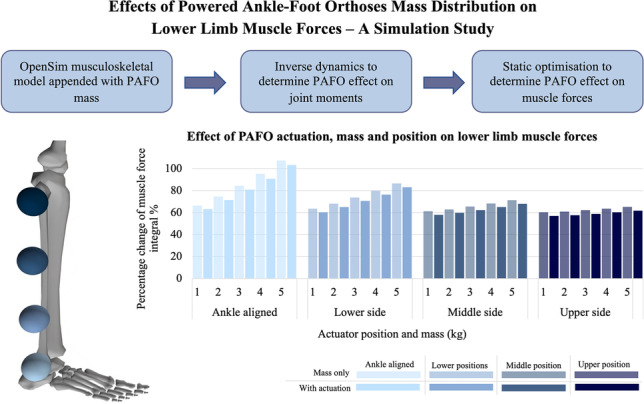

**Supplementary Information:**

The online version contains supplementary material available at 10.1007/s11517-023-02778-2.

## Introduction

The human gait cycle is a highly efficient and essential motion of human function; however, this gait cycle can be disrupted by injury, disease, or motor deficiencies which cause gait abnormalities [25]. To overcome the difficulties of pathologically affected gait, orthoses are commonly prescribed as to correct gait and assist in walking. These assistive orthoses are typically passive devices such as passive ankle–foot orthoses (AFOs). More recently powered orthoses and exoskeletons, which use motorized joints and power systems, have been developed to overcome gait abnormalities through active assistance [29]. These devices can be powered ankle–foot orthoses (PAFO) which assist in plantar and/or dorsiflexion of the ankle, powered knee or hip orthoses which control the flexion and extension of the knee and hip respectively, or combinations of these powered orthoses [29]. These devices can also be unilateral or bilateral, meaning they are fitted to one or both sides of the body, respectively. Currently, complete lower limb bilateral devices and powered knee orthoses are commercially available, while extensive research and development into PAFOs and unilateral devices continue [29].

Although passive orthoses are able to somewhat restore functional gait, they are largely limited to simply restricting joint range of motion, such as to prevent unintended plantarflexion in persons with foot drop [6]. In contrast to these devices, powered orthoses and exoskeletons are able to provide active assistance to joints, more effectively restoring motion and functional gait [31]. The design and efficacy of exoskeletons and powered orthoses are typically evaluated based on the improvement to kinematics and temporospatial parameters of gait [11, 27, 43]. However, as these devices have active components, they are inherently heavier than their passive counterparts, and therefore, this additional mass is expected to have some adverse effect on kinetics during walking [25, 28]. Furthermore, as these devices are typically analyzed in terms of their immediate effect on gait parameters, without much consideration for potential long-term effects, it is hypothesized that the additional mass would cause increased muscle forces, compensatory muscle activations, and possible muscular disuse, which could potentially have detrimental long-term effects [6, 16, 34].

As bilateral lower limb orthoses typically control multiple joints and support their own weight, the effect of orthosis mass is not as critical as for unilateral or single joint orthoses [29]. This is due to the fact that these unilateral powered orthoses require the user to support the weight of the device, especially during the swing phase of gait. PAFO devices require both the knee and hip joint to support and control the additional mass of the orthosis, and so these are of particular interest when considering the effect of the orthosis mass.

A common application for PAFOs is to overcome unintended plantarflexion and control dorsiflexion in persons who suffer from foot drop, which is commonly caused by nerve damage or hemiplegia such as after the occurrence of stroke [23]. Foot drop is usually a result of the paralysis of the peroneal nerve that innervates the dorsiflexor muscles, causing foot slap and toe drag [7, 23, 25, 26]. In persons with foot drop, the affected dorsiflexor muscles are the extensor digitorum longus, tibialis anterior, extensor hallucis longus, and peroneus tertius, which are all located on the anterior shank and cross the ankle joint [7].

It is known that additional mass added to the limb can increase the moment of inertia, contribute to gait asymmetry, and negatively impact the metabolic cost of walking [9, 25, 28]. However, the effect of the additional mass and mass distribution of an orthosis on lower limb muscle force and activity has not been systematically explored. Although studies have looked at the effect of both AFOs and PAFOs on muscle recruitment, demand, and metabolic cost of walking, typically only the effect of simulated ideal massless orthoses are considered [8, 30, 38]. While only one such study has considered the effect of PAFO mass on muscle force, this study is specific to children with cerebral palsy and crouch gait [30]. Thus, to our knowledge, no such research has looked at the effect of PAFO use on muscle demand and activity with consideration of both orthoses mass and mass distribution.

The aim of this research is therefore to identify the effect of PAFOs in terms of the orthoses effect on net joint moments and individual muscle forces, with consideration of the orthosis mass and mass distribution. It was hypothesized that through musculoskeletal simulation it would be possible to identify which muscles are most affected by PAFO mass and mass distribution. This could allow for the optimization of PAFO design as to minimize compensatory muscle forces and recruitment, more accurately mimic natural gait kinetics, and reduce unnecessary muscular disuse, potentially improving long-term effects of PAFO use.

## Method

### Musculoskeletal modelling

An open-source dataset from Camargo et al. containing lower limb biomechanics data and OpenSim models, scaled to the subject’s height and weight, was utilized in this study [10]. These lower limb models have 23 degrees of freedom and 92 musculotendon actuators, representing 76 muscles, adapted from widely used generic models [3, 4, 12, 42]. All muscles of the model are listed in Online Resource 1. The models have ball and socket joints at the hips, allowing hip flexion and extension, adduction and abduction, and internal and external rotation. The knee and ankle joints are modelled as hinge joints allowing for knee flexion and extension and ankle plantarflexion and dorsiflexion.

### Powered ankle–foot orthosis model

In order to simulate the effect of PAFO mass and mass distribution, the OpenSim models were modified for each subject to include the PAFO. While PAFOs are typically manufactured from very lightweight materials, such as carbon composites and thermoplastics [35], the actuator is typically the heaviest component of a PAFO. From the literature, it was deduced that this actuator contributes to an estimated 80% of the devices total mass [21, 29]. To represent this actuator positioned on the biomechanical model of the subject, 80% of the total device mass is included as a point mass positioned at the actuator center of mass (CoM). The remaining 20% of the orthosis mass is distributed across the shank brace and foot plate of the device. To represent this on the model, 10% of the total orthosis mass is distributed across the tibia component and the remaining 10% distributed across the calcaneus component, which is approximately the entire shank and foot segments of the OpenSim model.

**Fig. 1:**
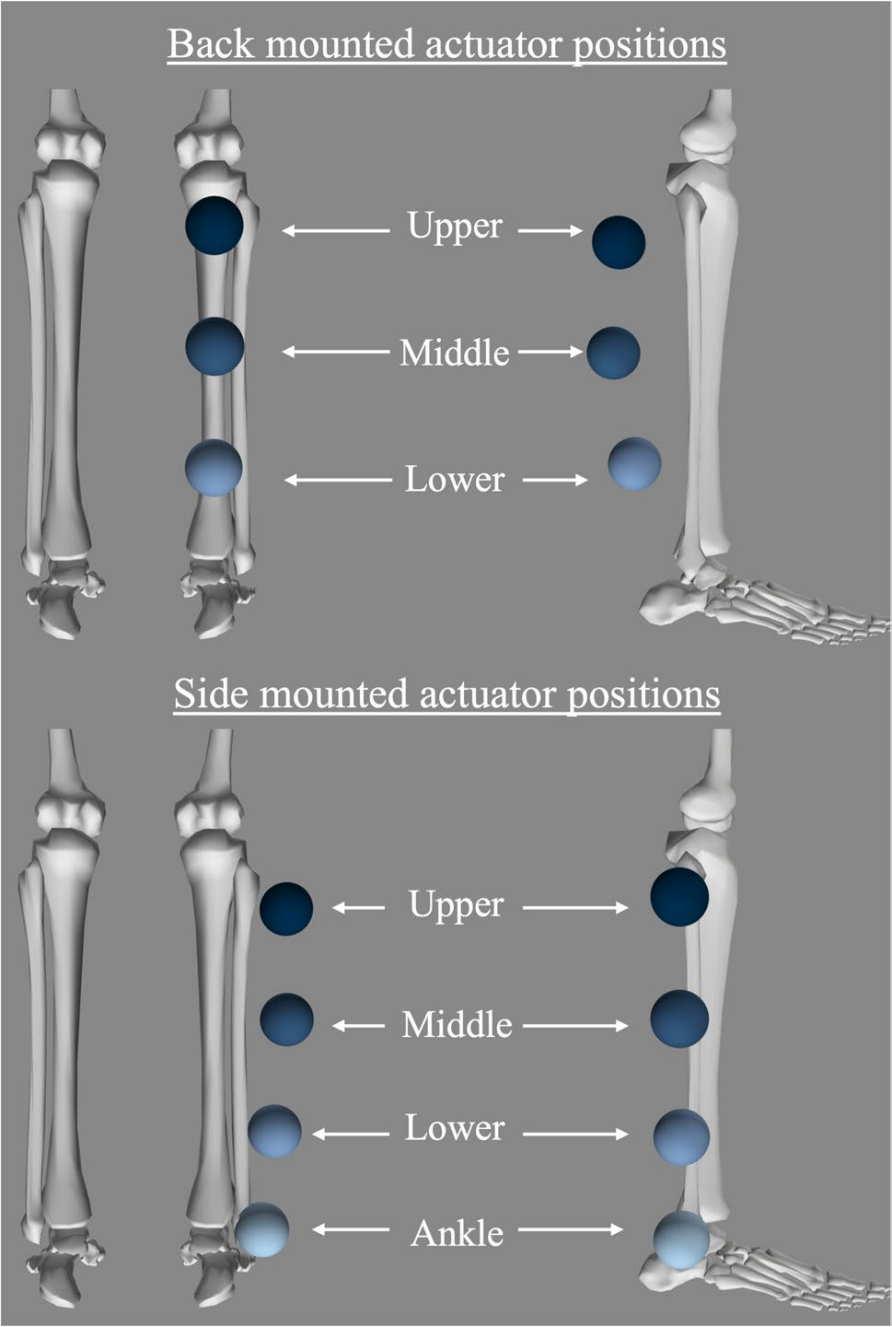
Possible actuator mass positions shown mounted on one of the subjects OpenSim models

While the basic shank brace and foot plate is typical of most PAFO designs, there are many different actuation technologies utilized and these can be positioned on multiple locations of the lower limb [1, 2, 15, 21, 25, 27, 29, 32, 41]. To analyze the effect of the different possible positions, as well as to identify the optimum position, seven different actuator positions were simulated, as illustrated in Fig. 1. The ankle aligned position is such that the actuator is positioned on the lateral side of the ankle in line with the joint axes. The lower, middle, and upper positions are offset distally from the knee joint and located at 75%, 50%, and 25% of the total shank length, respectively. Shank length was determined as the distance between the ankle and knee joint of the scaled models [39]. The side and back positions were such that the actuator CoM was positioned on the lateral and posterior side of the shank, respectively. The actuator was offset from the shank depending on user size, and this was determined by aligning the actuator CoM with surface markers attached to the OpenSim model. The actuator positions are summarized in Table 1 which also shows the notion used throughout this paper for each position. Furthermore, to identify effects of PAFO mass, five different orthoses masses were analyzed at each of the previously mentioned actuator CoM positions by adding additional weight to the model. The total PAFO masses explored were 1 kg, 2 kg, 3 kg, 4 kg, and 5 kg which are representative of many PAFO devices commonly found in the literature [1, 2, 15, 21, 25, 27, 29, 32, 41].

**Table 1:** Actuator positions

	Back mounted	Side mounted
Lower positions	Back mounted lower shank (L_B_)	Side mounted ankle aligned (A_S_) Side mounted lower shank (L_S_)
Middle positions	Back mounted middle shank (M_B_)	Side mounted middle shank (M_S_)
Upper positions	Back mounted upper shank (U_B_)	Side mounted upper shank (U_S_)

By modelling the PAFO with a point mass representing the actuator in multiple positions and for a range of different device masses, it is possible to model a PAFO for dorsiflexion assistance which is representative of many different PAFO designs without restricting this study to one such design. Furthermore, through this method of simulating the PAFO as a distributed mass, rather than fitting subjects with an actual device, it is possible to determine the effect of the mass and mass distribution only, without the PAFO restricting the motion of the ankle and affecting gait kinematics. This point mass simulation approach is a simplified and more efficient method which produces the same results as compared to modelling the PAFO as a rigid body which does not hinder motion.

### Data

From the open-source biomechanics dataset from Camargo et al., kinematic and ground reaction force (GRF) data was used. This data was captured from 32 motion capture markers recorded at 200 Hz (Vicon. Ltd., Oxford, UK), positioned as per the gold standard Helen Hayes Hospital marker set, and from force plates of an instrumented treadmill recorded at 1000 Hz (Bertec, Ohio, USA) [10]. This marker set allows for excellent tracking of the lower limbs during ambulation but the lack of upper limb markers restricts the ability to model upper body motion. However, lower limb data was adequate for this study as upper limb motion does not significantly contribute to propulsion in gait such that omitting this data would have negligible effect on results [17, 18, 36]. This data was obtained from 10 healthy adult subjects, 4 females and 6 males, of height 1.71 ± 0.06 m, mass 68.03 ± 12.90 kg, and age 21.1 ± 3.57 years. This study used only 10 of the available 20 subjects as this provided an adequate range of subjects in terms of height and weight, without additional computation time. Further details about these subjects are outlined in Table 2.

**Table 2:** Subject information

Subject	Gender	Age (years)	Height (m)	Weight (kg)
1	M	20	1.80	74.84
2	F	21	1.63	63.50
3	M	19	1.73	58.97
4	M	21	1.78	96.16
5	F	20	1.65	55.79
6	M	19	1.68	61.23
7	M	19	1.70	68.04
8	F	21	1.73	72.57
9	F	20	1.63	52.16
10	M	31	1.77	77.03
		21.1 ± 3.57	1.71 ± 0.06	68.03 ± 12.90

A representative single gait cycle for each of the 10 subjects was obtained from the data by averaging the kinematic marker and GRF data of all full gait cycles which were completed when the treadmill was set at a walking speed of 1.25 m/s. This walking speed was chosen as it is within a range of self-selected walking speeds typical of a person with gait impairments, while maintaining enough speed such that this would be considered a successful gait cycle with the PAFO assistance [24]. Across the 10 subjects, there were at least 31 full gait cycles available at this walking speed. By utilizing the treadmill data, it was possible to get complete and consecutive gait cycles at this steady speed and with corresponding GRF data.

### Inverse kinematics and inverse dynamics

Using the averaged kinematic marker data, inverse kinematics was performed for each subject using the OpenSim 4.2 inverse kinematics tool [13, 33]. This calculates the joint angle trajectories required to achieve the recorded gait motion. These results are then considered the ideal gait kinematics, which is aimed to be achieved with the PAFO and is then used for the proceeding inverse dynamic and static optimization analyses. By using these ideal gait kinematics, we are assuming that the hypothetical PAFO is able to perfectly restore normal gait, which while this is not currently possible, is the ultimate goal for PAFOs.

The net joint moments and forces acting at each of the joints in the model throughout the gait cycle are calculated by utilizing the OpenSim 4.2 inverse dynamics tool. This solves the equations of motions when the model is defined by the generalized coordinates, accelerations, and velocities, previously calculated through inverse kinematics [5]. These equations of motion are outlined in Online Resource 2.

With the addition of the GRFs, inverse dynamics is able to calculate the internal net joint moments and forces at each timepoint throughout the gait cycle. Therefore, to identify the effect of the PAFO mass and mass distribution on net joint moments, inverse dynamics is performed for each subject without the PAFO and with each combination of actuator position and PAFO mass.

Although the joint angles and net joint moments can be determined and analyzed for all degrees of freedom of the hip, knee, and ankle, the focus of this study was primarily on the sagittal plane. This is due to the sagittal plane being the most critical plane of movement for human locomotion. Therefore, throughout this paper, all mention of joint angles and moments will refer to that of the sagittal plane.

### Static optimization

The OpenSim 4.2 static optimization tool is then utilized to identify the individual muscle forces required when walking with a PAFO. Although both the OpenSim static optimisation and computed muscle control (CMC) algorithms can be used to estimate muscle force and activation, static optimization was preferred in this study due to its efficiency and accuracy in the case of human locomotion, and due to CMC having a tendency to overestimate muscle forces [19, 22, 38]. Further to the inverse dynamics tool, the static optimization tool calculates the individual muscle forces at each time interval as defined by the GRFs and generalized coordinates, accelerations, and velocities, identified by inverse kinematics. The static optimization algorithm calculates the individual muscle force by minimizing the sum of squared muscle activations, subject to each muscle’s force–length-velocity characteristics [5]. The equations used are depicted in Online Resource 2. Therefore, this tool is able to estimate the optimum muscle forces required to achieve the defined motion. Hence, when the PAFO is appended to the model, the effect that the actuator mass and its position of attachment has on individual muscles can be identified. 

In order to reduce the effect of modelling and marker data errors, the OpenSim 4.2 residual reduction algorithm was utilized, which minimizes the reliance on non-physical compensatory forces during static optimization [5]. The torso CoM was optimized for each model using the residual reduction algorithm tool, and while residuals were minimized as best as possible, some larger residuals were observed. These residuals were attributed to the lack of upper body motion data, which was not available to be modelled. While there were some discrepancies with the residual forces and moments, there was very minimal marker error, aligning with best practice recommendations [5, 20]. The minimal marker error was due to the gait data being averaged for each subject and then further filtered. Therefore, after the residual reduction algorithm was used, the unadjusted kinematic data was used with the optimized models for static optimisation.

As to include the assistive force of the PAFO in the static optimization analysis, a reserve actuator has been appended to the model and is modelled as an ideal actuator acting in the sagittal plane across the ankle joint. This actuator only assists in the dorsiflexion motion and does not affect plantarflexion motion, such that it would be an ideal PAFO for persons affected by foot drop. By adjusting the weighting of the optimal torque value to 1MNm, this reserve actuator has a negligible penalty on the static optimization cost function, therefore allowing for the simulation of dorsiflexor muscle weakness and ideal dorsiflexion assistance by the PAFO [30, 38]. Through this method, static optimization can be completed such that it is possible to identify the muscle forces required to achieve the ideal gait cycle when the person suffers from foot drop and uses the PAFO.

To identify the effect of each of the PAFOs mass, mass distribution, and assistive force, muscle forces were calculated using the static optimization tool for multiple situations. The following situations were simulated for each subject and all combinations of PAFO mass and actuator positioning:

- without the PAFO (no mass and no actuation)
- with PAFO active but no mass (actuation only)
- with PAFO worn but not active (mass only)
- with PAFO worn and active (actuation and mass)

While these inverse dynamics and static optimization approaches are useful to simulate both the effect of the orthosis mass and position on net joint torques and forces, and individual muscle forces and activations, the additional mass of the PAFO is not considered in the GRFs used. However, as all simulations are completed with the same assumption that the GRFs have not changed, comparisons between the different masses and actuator positions can still be made. Furthermore, the kinematic data used is that of the subject’s average healthy gait cycle and the effect of the additional mass of the orthosis or the potential effects of foot drop is not considered in this motion. Therefore, this kinematic data is assumed to be the ideal motion which is aimed to be achieved with the use of an ideal PAFO. As such, the inverse dynamic and static optimization analyses determine what net joint torque and forces, and individual muscle forces and activations are required by the subject with the use of the PAFO, in order to achieve this ideal gait.

### Data analysis

To quantify how the PAFO affects net joint moments and muscle forces, the results for each actuator position, mass and actuation situation was compared against the results of the unassisted and with no added weight situation. First, the results from inverse dynamics and static optimization were normalized to each subject’s body mass, as to allow for comparison across the range of different sized subjects. These results were then averaged across the 10 subjects to find the mean net joint moments and individual muscle forces for each of the different actuator positions, masses, and actuation situations. Through doing so, it is possible to identify the expected effect of the PAFO mass and actuation position on a wide range of persons. From this, averaged joint angle and moment curves were created, as well as average muscle force curves for all 31 right lower limb muscles with each different PAFO situation.

For the net joint moments, the peak extension and peak flexion moments for each joint were identified from the averaged data. To identify how much the PAFO mass and actuator position affected these average peak net moments, the percentage change between the peak net moment of the no PAFO and each PAFO situation was calculated. The percentage change formula is shown in Eq. 1, where ${V}_{1}$ is the no PAFO value and ${V}_{2}$ is the value for each PAFO situation.

1 $$Percentage\;change= \frac{({V}_{2}-{V}_{1})}{|{V}_{1}|} \times 100$$

In terms of the individual muscle forces, to compare the no PAFO situation to the added mass and different positions, as well as with and without actuation, the integral of the muscle force curve was calculated for each muscle and situation [7, 30]. Equation 2 depicts the formula for the muscle force integral across the gait cycle, where *F* is the muscle force curve [7]. This integral is used to identify the effect across the entire gait cycle and was computed using trapezoidal integration in MATLAB 2021a.

2 $$Muscle\;force\;integral= {\int }_{i=1}^{100}\left|{F}_{i}\right|dGait\;cycle\;\left[\%\right]$$

Furthermore, the percentage change was calculated for the muscle force integral of each muscle, as per Eq. 1, to identify the difference between the no PAFO situation and the various PAFO masses and actuator positions. The overall effect was also calculated by averaging the percentage change of the muscle force integral across all muscles of the right lower limb, with the omittance of the muscles affected by foot drop and lower back muscles, as to identify the PAFO effect on all other muscles. Therefore, it is possible to identify the effect that the PAFO mass, position, and actuation have on both the individual and overall muscle forces throughout the lower limb.

## Results

### Joint angles and moments

**Fig. 2:**
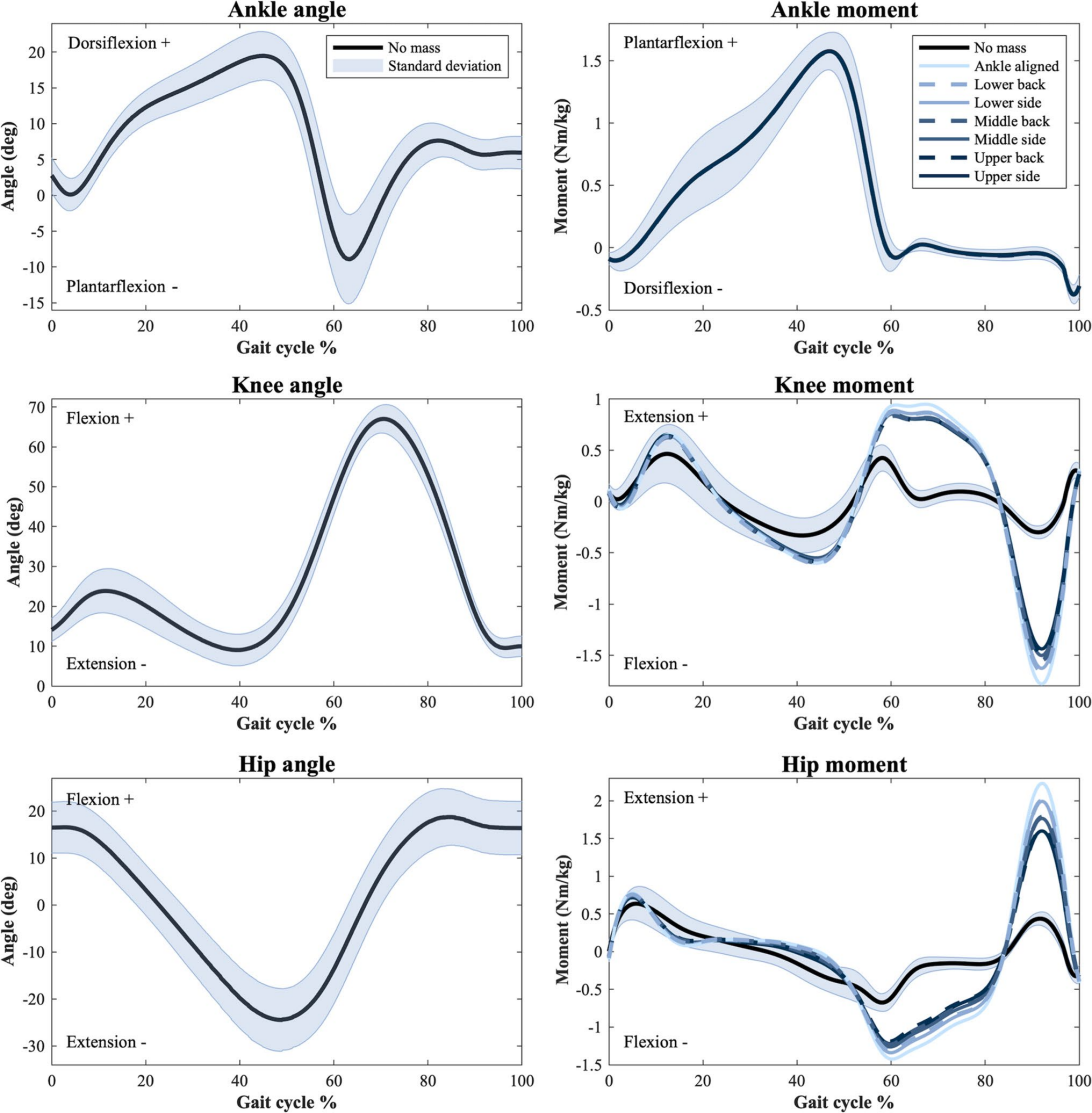
Averaged joint angles and net joint moments across the gait cycle. For the joint moments, the different actuator positions are shown for the 3 kg PAFO mass

The averaged joint angles and moments produced by inverse kinematics and dynamics are shown in Fig. 2. This shows the net joint moment for each actuator position for the 3 kg situation, and these results are further detailed in Online Resource 3, which depicts the peak percentage change from the no PAFO situation and each different mass and actuator position.

For the ankle joint, there is negligible change to the net joint moment across the gait cycle and as such there is no change seen in Fig. 2. However, the knee and hip joint flexor and extensor moments are clearly much more affected by the additional PAFO mass as well as by the actuator positioning, particularly in the swing phase, from 60 to 100% of the gait cycle. Figure 2 shows that lower positions (L_B_, L_S_, and A_S_) have an increased effect on both the knee and hip, flexor and extensor moments compared to higher positions, with the side and back positions having very similar results. For the percentage change of the knee peak joint moment, there was an average increase of 27.01% and 110.91% between the average of the upper positions and the A_S_ position, for the extensor and flexor moments, respectively. Furthermore, there was an average increase of 147.10% and 34.67% between the average of the upper positions and the A_S_ position, for the hip extensor and flexor moments, respectively.

### Muscle forces

**Fig. 3:**
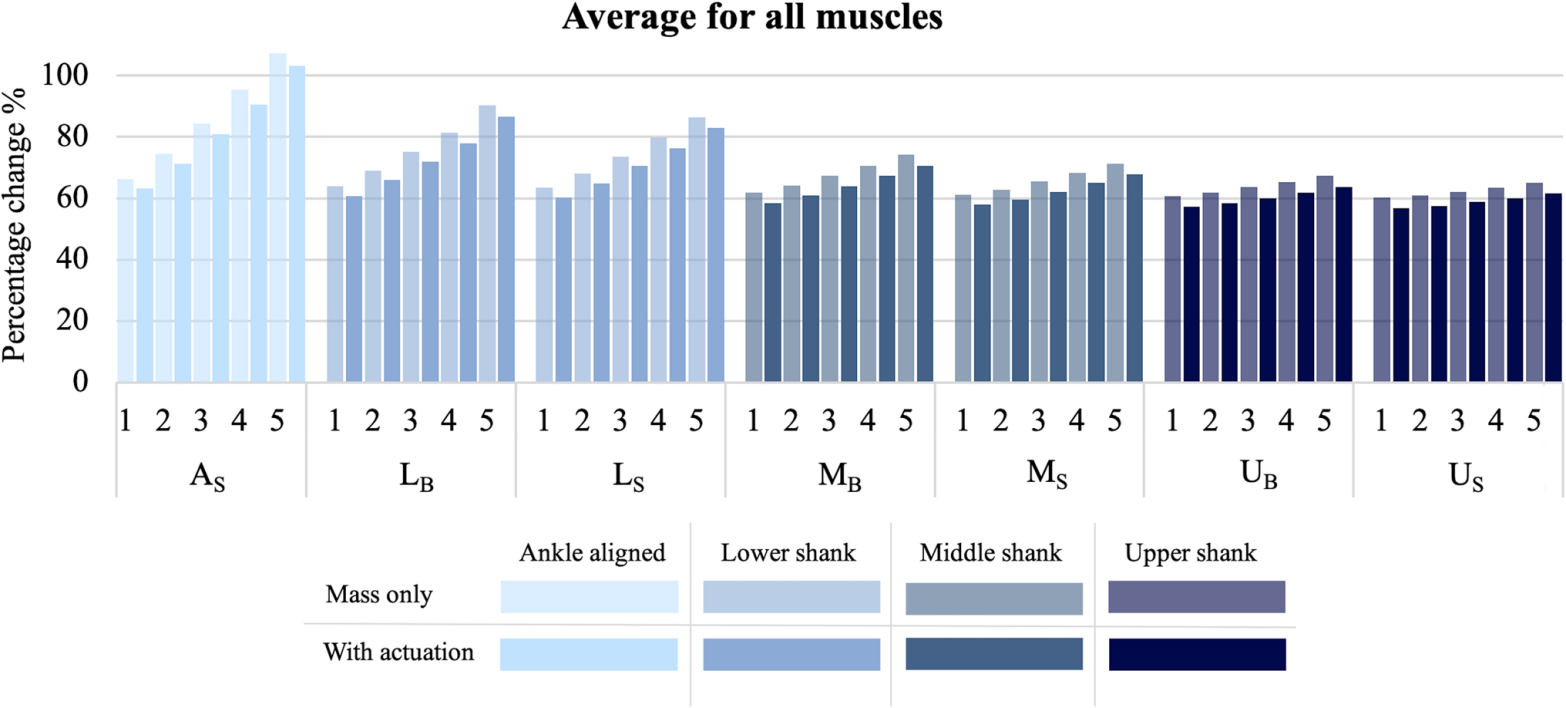
Percentage change of the muscle force curve integral, comparing each actuator position, PAFO mass, and with or without actuation, for the average of all lower limb muscles

Figure 3 depicts the percentage change of the muscle force integral for the average of all muscles of the right lower limb. From this figure, the average effect of the actuator position can be clearly seen, where the percentage change increase for all masses with actuation were as follows: 81.79% for A_S_, 72.62% for L_B_, 70.97% for L_S_, 64.28% for M_B_, 62.52% for M_S_, 60.22% for U_B_, and 59.00% for U_S_. Similar trends were seen for the no actuation situation but with further increased percentage change values. This shows that there is an increase in muscle force due to the addition of the PAFO, with the overall muscle forces being most negatively affected when the actuator is positioned lower on the limb and posteriorly.

Furthermore, Fig. 3 demonstrates that increased mass of the PAFO results in an average increase in muscle force throughout the limb. However, this increased mass has a greater effect when the actuator is positioned lower on the limb. In comparison to when only the mass is added, it can also be seen that there is an overall reduction in muscle force throughout the lower limb when the actuator is utilized. While the majority of the muscles align with these trends, muscles which did not behave in this way due to PAFO mass or actuation have been identified in Table 3.

**Table 3:** Effect of PAFO mass and actuation on muscle force

Effect of PAFO	Affected muscles
Reduced muscle force with the additional PAFO mass	Flexor digitorum longus Flexor hallucis longus Peroneus brevis Peroneus longus Soleus Tibialis posterior
Reduced muscle force with heavier PAFO masses	Adductor magnus Peroneus brevis (at upper and middle positions only) Rectus femoris (at M_B_ and U_B_ positions only) Vastus intermedius (except at A_S_) Vastus lateralis (except at A_S_) Vastus medialis (except at A_S_)
Increased muscle force with active actuation compared to mass only	Adductor brevis Adductor magnus Flexor digitorum longus Flexor hallucis longus Gluteus maximus Lateral gastrocnemius Medial gastrocnemius Peroneus brevis
Negligible change between mass only and with active actuation	Adductor longus Pectineus Rectus femoris Vastus intermedius Vastus lateralis Vastus medialis

**Fig. 4:**
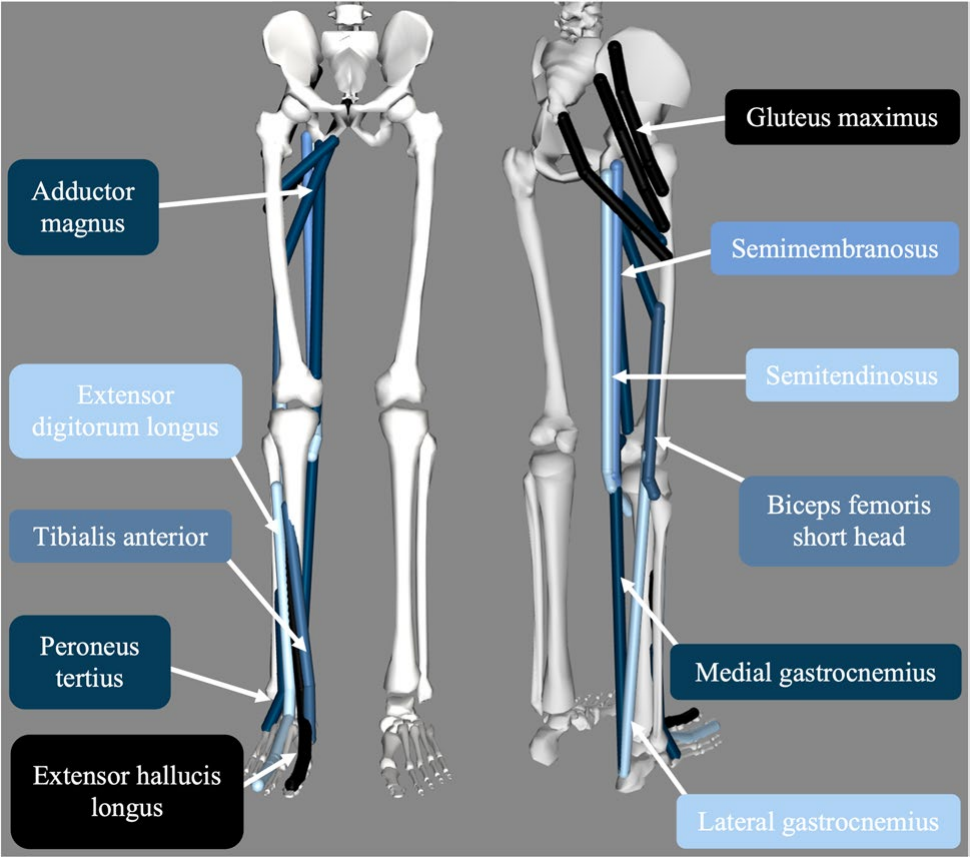
OpenSim model showing muscles affected by the PAFO and muscles affected by foot drop

From the 31 muscles that were analyzed, there were seven muscles identified as the most affected by the addition of the PAFO. These were the adductor magnus, gluteus maximus, lateral and medial gastrocnemius, semitendinosus, semimembranosus, and the biceps femoris short head. These seven muscles are shown on the OpenSim model in Fig. 4. Furthermore, this figure shows the four dorsiflexor muscles affected by foot drop which the actuator compensates for; extensor digitorum longus, extensor hallucis longus, tibialis anterior, and peroneus tertius. The gluteus maximus and adductor magnus are each represented as three separate musculotendon actuators as they span large areas; however, their muscle forces were combined to find the total muscle force of these muscles.

**Fig. 5:**
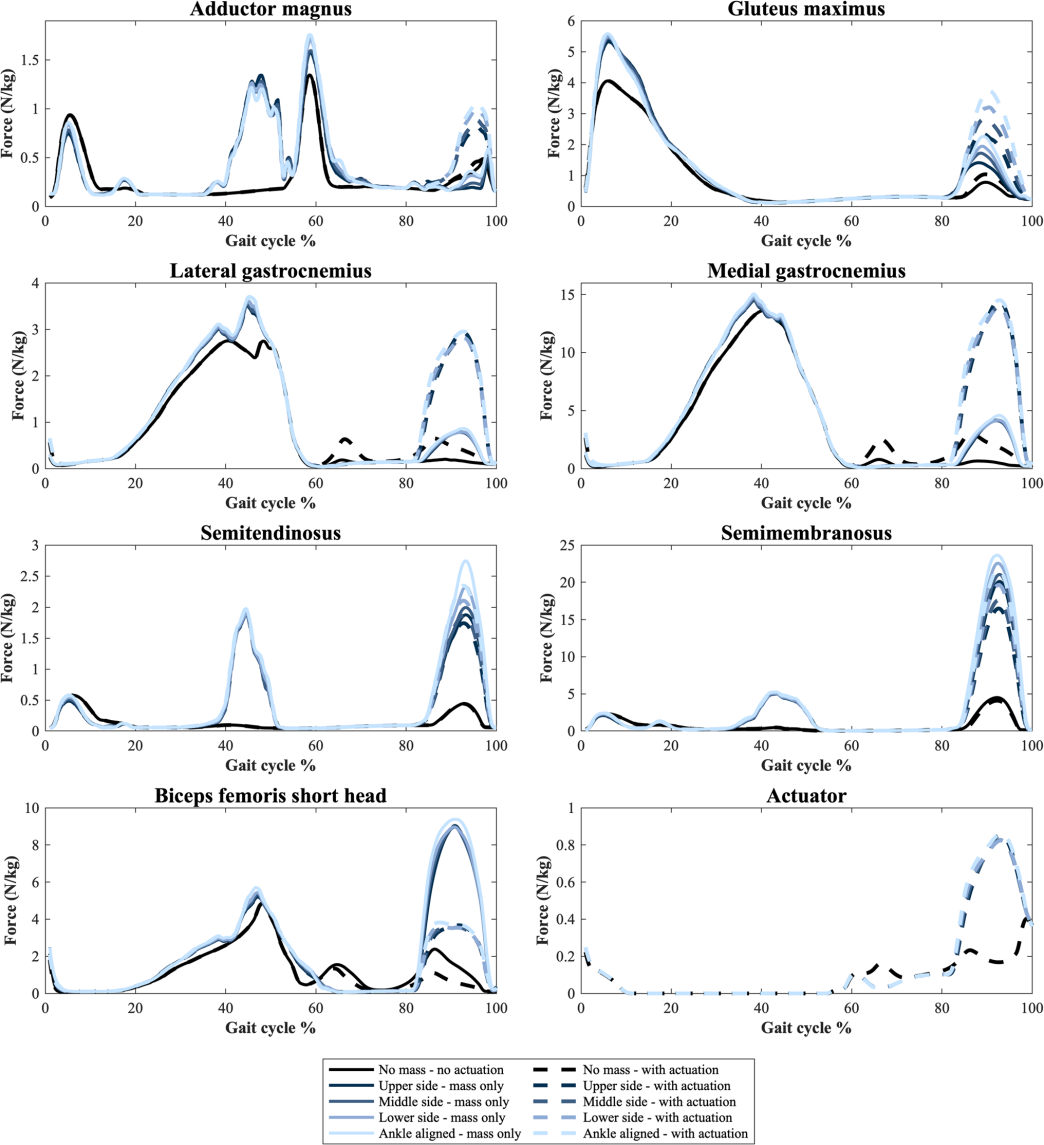
Muscle force curves for each of the muscles identified as most affected by the PAFO as well as the actuator force curve. Each actuator position is shown for the 3 kg PAFO mass

For the most affected muscles, the muscle force curves are shown in Fig. 5, where the back positions have been omitted and only the 3 kg situations have been included for ease of observation, as the side and back mounted positions have similar results which are not easily recognizable. This figure also includes the force curve for the actuator, showing when in the gait cycle the PAFO is actively taking over from the affected muscles.

**Fig. 6:**
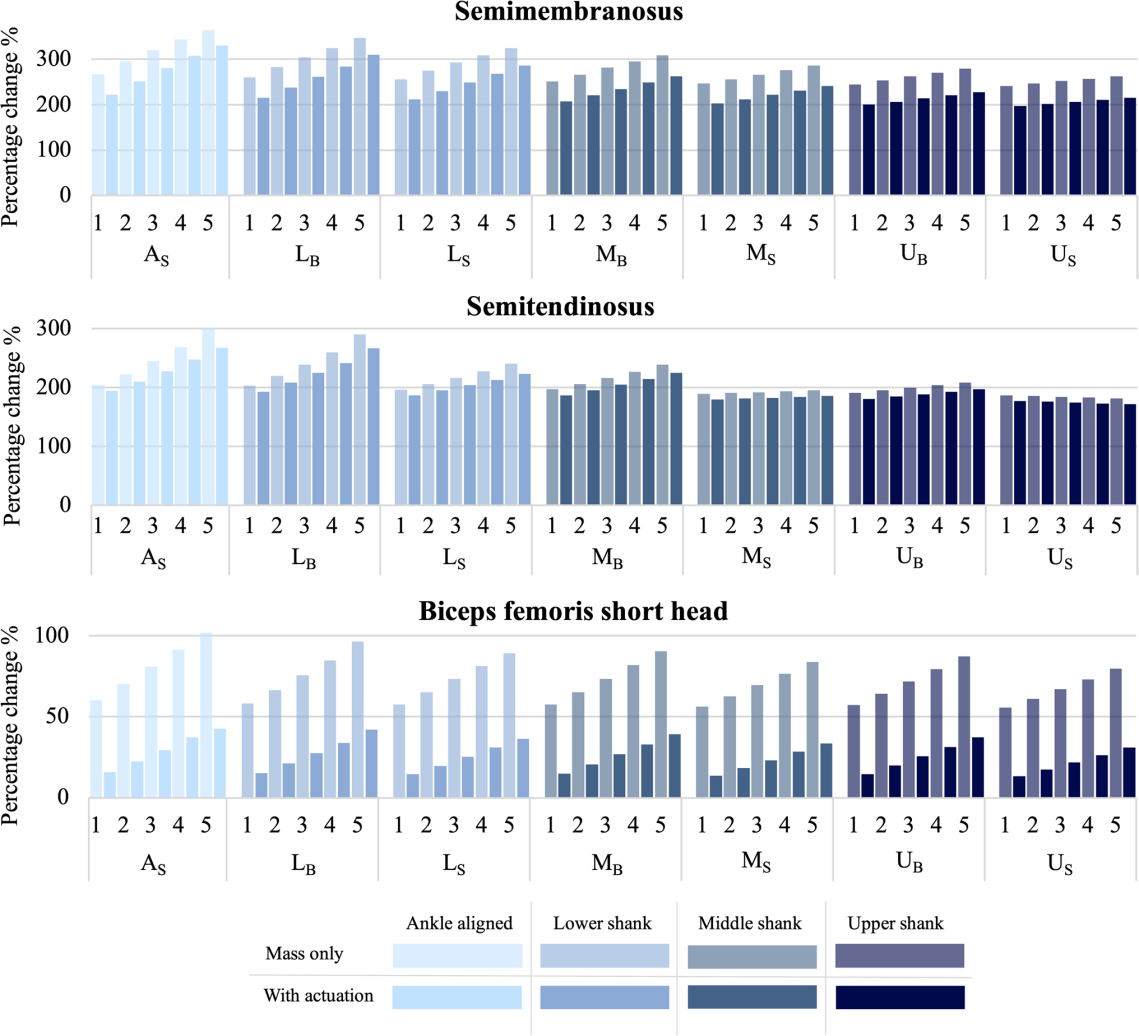
Percentage change of the muscle force curve integral, comparing each actuator position, PAFO mass, and with or without actuation, for the semimembranosus, semitendinosus, and bicep femoris short head

Of the seven muscles identified as the most affected by the PAFO, three of these muscles had a reduced muscle force when the actuation was active. These were the semitendinosus, semimembranosus, and the biceps femoris short head. The percentage change of the muscle force integral for these muscles is shown in Fig. 6. These muscles belong to the hamstring group and are all involved in knee flexion, with the semitendinosus and semimembranosus also acting on hip extension. Each peak in force for these hamstring muscles, as seen in Fig. 5, aligns with points of maximum knee flexion moments, seen in Fig. 2. From both Figs. 5 and 6, it can be seen that the mass only situation has a much greater effect on the muscle force for these muscles, particularly the biceps femoris short head. Figure 6 also shows that the lower positions each have a greater impact on the muscle force than higher positions and side positions are slightly less affected than back positions. At each position, the increase in mass has a negative effect on the muscle force with an increased percentage change seen. However, for the semitendinosus at the M_S_ and U_B_ positions, the increase in percentage change is only very slight and at the U_S_ position a decrease in the percentage change is seen as the mass is increased.

**Fig. 7:**
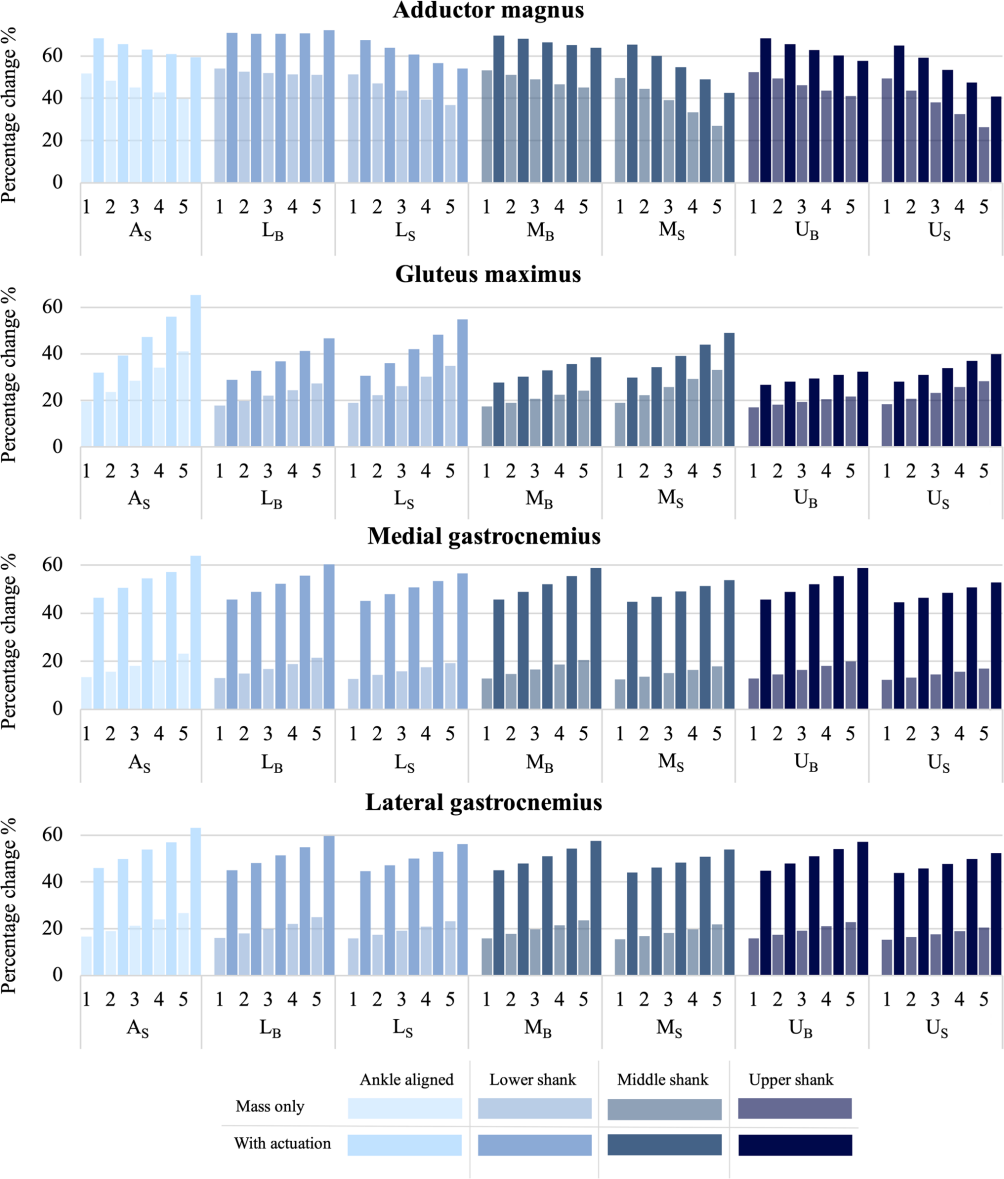
Percentage change of the muscle force curve integral, comparing each actuator position, PAFO mass, and with or without actuation, for the adductor magnus, gluteus maximus, medial gastrocnemius, and lateral gastrocnemius

Of the most affected muscles, the remaining four muscles each had an increased muscle force and percentage change of the muscle force integral when the actuator was active. These muscles are the adductor magnus, gluteus maximus, and the lateral and medial gastrocnemius. The percentage change of the muscle force integral for these muscles is shown in Fig. 7. The adductor magnus is the largest muscle in the medial compartment of the thigh and is responsible for both flexion and extension of the hip, as well as adduction. As seen in Fig. 5, the adductor magnus has multiple peaks in the muscle force which are affected by the addition of the PAFO. Peaks at 45% and 60% of the gait cycle align with points of hip flexion and these are more negatively affected by the additional mass. There is also a peak observed at 90% of the gait cycle which aligns with hip extension and at this point, the actuator causes a much greater increase in force, as compared to the mass only situations. Shown in Fig. 7, the adductor magnus consistently has a decrease in the percentage change of the muscle force integral when the mass is increased, for both the mass only and actuation situations. There is an exception to this decrease for the L_B_ position with actuation, which is seen to be steady for 1–4 kg but has a slight increase at 5 kg. Figure 7 also shows that the side positions with higher masses are much less affected than the same masses with the actuator positioned at the back of the limb. Furthermore, the A_S_ position is much less affected than the L_B_ position and only slightly more affected than the L_S_ position.

The gluteus maximus is the most superficial of the gluteal muscles and acts on hip extension and propulsion during walking. The peaks in gluteus maximus force, seen in Fig. 5, align with increased hip extension moments during the gait cycle. The peak observed at 90% of the gait cycle is due to the hip extension moment in preparation for heel strike, and at this point, the additional mass causes an increase in muscle force; however, this is increased even more with the PAFO actuation. Figure 7 shows that for the gluteus maximus, the increased mass consistently causes an increase in the percentage change of the muscle force integral, and that the lower positions are more affected than the higher positions, and side mounted positions are slightly more affected than back mounted positions.

The gastrocnemius muscle, made up of the lateral and medial heads, are part of the triceps surae group which are responsible for plantar flexion of the ankle and propulsion during walking. The gastrocnemius muscle also plays a role in knee flexion as it is a biarticular muscle crossing the knee and ankle joint. For the lateral and medial gastrocnemius, the force patterns and percentage change trends are very similar with the medial head exhibiting a greater force. In Fig. 5, the initial peak, seen at approximately 40% of the gait cycle, aligns with the stance phase plantarflexion moment of the ankle, with the additional mass slightly increasing the muscle force at this point. The large peak at the end of swing phase aligns with the knee flexion moment in preparation for heel strike as well as the increased dorsiflexion actuator force. At this point, the active actuator situation causes a large increase in muscle force. This can also be seen in Fig. 7, where the active actuation situation has a much more significant impact on the muscle force than the mass only situation, across all positions and masses. It can also be seen that the increased mass causes an increased effect on the muscle force and the lower actuator positions are slightly more affected than those positioned higher up. At each mass, the back positions are slightly more affected than the side positions, for both the lateral and medial gastrocnemius.

## Discussion

Existing PAFO design evaluation typically considers only how the device improves kinematics and temporospatial parameters of gait, and the effect on net joint moments or individual muscle forces is not well explored [27, 43]. Furthermore, the few studies which do explore the effect of PAFOs on moments and muscle forces typically do not consider the mass of the device [8, 30, 38]. While these kinematic and temporospatial parameters are important factors in achieving successful gait with the PAFO, there are potential long-term negative effects caused by changes to net joint moments, individual muscle forces, and activity, which are largely attributable to the device mass. In terms of net joint moments, it is believed that long-term increased loading of joints is likely to contribute to degradation of the joint, potentially leading to osteoarthritis [23]. For muscles that are recruited to compensate for the additional PAFO, such that they have a greatly increased muscle force, muscular fatigue may occur which can result in the inability to achieve a successful gait, pain, and increased metabolic rate and exhaustion [9, 37]. Furthermore, muscles which experience a significant reduction in force due to the addition of the PAFO could have detrimental effects due to muscle disuse [6, 34]. This can cause muscle atrophy and neural circuit degradation which may eventually result in loss of muscular control [6, 16]. Therefore, the effect of PAFO mass distribution on net joint moments and individual muscle forces must be considered to identify significant effects of the PAFO and to prevent long-term negative effects through improved PAFO design.

Referring to Fig. 2, for normal walking with no added weight, the joint angles and moments align with studies throughout the literature [23, 39, 40]. As the joint kinematics used throughout this study are for the same ideal motion, the joint angles do not change with the addition of the PAFO mass. However, this additional mass results in clear changes to the net joint moments, particularly for the knee and hip joint. This is due to the increased mass of the entire lower limb requiring additional net joint moments to control the movement, particularly when the mass of the limb is no longer supported by the ground, as in swing phase.

As there is only 10% of the total PAFO mass appended to the foot segment, representing the foot plate, the difference in the net joint moment for the ankle was negligible, as this joint does not need to control much additional mass. In terms of the knee and hip joint moments, it was found that the lower positions, particularly the A_S_ position, are much more negatively affected by the additional PAFO mass compared to the higher positions. This is due to the displacement of the lower limb CoM and thus the change in inertia of the limb, particularly during swing phase. When the additional mass of the PAFO is positioned more distally on the limb, the CoM of the entire lower limb complex is shifted further from the hip and knee joint, thus causing the moment of these joints to increase. This is similar to findings by Browning et al. and explains why the higher up actuator positions require lower joint moments as compared to the lower positions [9].

The net joint moments were found to be consistently more affected by actuator masses being positioned more distally on the limb compared to more proximal positions, with the lower positions increasing the percentage change by an average of 27.09% across all peak joint moments, in comparison to the upper positions. However, the effect of the actuator being mounted on the back or side of the leg, irrespective of the distal position, has varying effects for each joint moment with no consistent pattern. The side mounted actuator positions were found to be slightly better for the knee flexor and the hip extensor moments, whereas the back mounted position was slightly better for the knee extensor and hip flexor moments. This is likely due to the point in the gait cycle that these moments are occurring and the inertia of the limb at this time.

In terms of the overall muscle forces of the lower limb, the muscles were least affected by the actuator being positioned higher on the limb and in side mounted positions, with lighter total PAFO masses and when the actuator was active. As there were similar trends seen for the actuation and no actuation situation, it can be concluded that the increase in muscle forces is primarily attributable to the mass and position of the device, with the actuation slightly reducing these forces.

While these trends were seen for the overall average muscle force, not all individual muscles behaved this way and the effect of the PAFO had varying results. Interestingly, the muscles which had a reduced force due to the additional mass of the PAFO were the plantar flexor muscles, excluding the gastrocnemius. As the gastrocnemius is the only plantar flexor that is biarticular, also acting on the knee, this suggests that the increased force of the gastrocnemius is likely due to the additional force required at the knee joint, rather than the ankle. Muscles which had an increased muscle force when the additional mass was lighter in weight were mostly the quadriceps and adductor magnus muscles, particularly when the mass was at higher positions. For these muscles, the increase in force was at the push-off and toe-off stage, which suggests that heavier masses that are positioned higher reduce the force required because the shift in inertia helps to begin the swing phase. Finally, the muscles which had an increased force with PAFO actuation were two of the adductor muscles, some of the plantar flexor muscles and the gluteus maximus. It can be deduced that the reason for the increase in muscle force with actuation for these muscles is due to the flow-on effect of the PAFO and how the activity of certain muscles affects each other, which is further discussed below.

Furthermore, similar to the net joint moments, lower positions were found to cause a more significant effect on individual muscle forces, and side and back positions had varying results. This is again related to the displacement of the lower limb CoM and the change to the moment of inertia, requiring more muscular control when the PAFO mass is positioned lower on the limb. For the individual muscles analyzed, it was evident that for most muscles, the back positions were more affected than the side; however, the opposite was true for the gluteus maximus. The side mounted position likely affects the gluteus maximus more significantly due to the lower limb CoM being offset more anteriorly in the sagittal plane, such that the gluteus maximus requires more force to achieve hip extension.

In terms of the individual muscles analyzed, it was found that while the additional mass causes an increase in muscle force for all these muscles of interest, the addition of the PAFO actuation causes varying effects to these muscles. These varying effects can be attributed to the antagonistic pairing of muscles in the lower limb, as well as the fact that multiple lower limb muscles are biarticular and that the activity of certain muscles can have flow-on effects to other muscles.

The muscle force of the gastrocnemius is found to have increased in the swing phase of gait, which is believed to be to assist in knee flexion while also opposing the dorsiflexion force of the actuator. As an ideal actuator has been simulated which provides the required force to achieve the ideal gait defined by the kinematics, the gastrocnemius is recruited to oppose this force in the end of swing phase, as the position of the ankle is to be maintained. Thus, the gastrocnemius and the actuator act as an antagonistic pair, opposing each other’s force to maintain the angle of the ankle. Therefore, the large increase in the gastrocnemius force is seen when the actuator is active.

The hamstring muscles have an increased force with the additional mass; however, a reduction in force with actuation is seen which is thought to be due to the increased force observed in the gastrocnemius. As the gastrocnemius is a biarticular muscle, acting on both the ankle and knee joint, the large force observed due to it acting to oppose the actuator also further contributes to knee flexion. Therefore, the hamstring muscles require less force to flex the knee during this swing phase of gait, such that with actuation a reduction in hamstring muscle force is seen. This is particularly true for the biceps femoris as this muscle acts only on knee flexion.

Reduction in hamstring muscle force then has a flow-on effect to the gluteus maximus. As the semimembranosus and semitendinosus have reduced force when actuation is active, their reduction in force also affects the hip extension moment as these muscles are biarticular, acting on both the knee and hip joint. Therefore, the gluteus maximus must increase its force to achieve the required hip extension moment, as to maintain the position of the hip in the terminal swing phase. Furthermore, as the gluteus maximus must increase its force to maintain the position of the hip, the adductor magnus, acting as an antagonistic pair with the gluteus maximus, must increase its force just before heel strike as to control hip flexion and prepare for heel contact. Therefore, this flow-on effect shows how individual muscles can be more significantly affected by the addition of the PAFO, due to antagonistic pairs and biarticulate muscles, as their activity consequently affects other muscles.

Conclusively, while there are varying results for the different PAFO situations on net joint moments and individual muscles throughout the lower limb, in order to optimize the PAFO design in terms of these moments and muscle forces, it is suggested that the majority of the PAFOs mass should be positioned on the lateral side of the leg, more proximally toward the knee, and the mass should be kept as lightweight as possible. For this study, this is the side mounted upper shank position (U_S_) and with a total PAFO mass of 1 kg. However, while this PAFO situation is shown to reduce the overall lower limb muscle forces compared to other situations, the variation in effects on individual muscles, as well as the flow-on effects of muscles on each other, poses the issue of potential long-term detrimental effects of increased muscle forces or muscular disuse. For a person with foot drop such that only the four dorsiflexor muscles are affected, this may not be a significant issue. However, for sufferers of stroke, hemiplegia, or other neurological conditions, where multiple muscles may be affected by partial paresis or weakness, this may pose a significant risk of further deterioration and negatively impact rehabilitation.

Furthermore, while this simulation study has assumed that ideal gait kinematics are achieved with the PAFO, it is known that altered gait kinematics and temporospatial parameters would be expected due to foot drop and the addition of the PAFO [6, 14, 23, 25, 40]. This altered gait is often a result of compensatory gait patterns that allow the person to achieve successful locomotion in the most efficient way, accounting for the restrictions imposed by foot drop or the PAFO. Thus, these compensatory strategies may also further result in changes to muscle forces and recruitment, potentially resulting in further detrimental effects [6, 7, 34, 40]. Therefore, it is suggested that PAFO design should consider each individual person’s current gait, lower limb muscular capabilities, and pathological condition in order to optimize PAFO design to prevent these potentially detrimental effects. Biomechanical analysis and static optimization could be used as a design tool to allow for patient-specific optimization to ensure the PAFO mass distribution complements their individual gait, avoiding potentially detrimental muscle forces and muscular disuse.

Although altered kinematics and temporospatial parameters would realistically occur, by utilizing the gait data of healthy persons and simulating affected dorsiflexors and an ideal actuator, it was possible to isolate the effect of only the device assistance, mass, and mass distribution from other variables, such as device stiffness, control and power, psychological factors, or compensatory gait patterns. Moreover, while it is suggested to consider an individual person’s gait and capability, the methods used in this study allows for the effect of PAFO mass and mass distribution to be estimated in a generally predictive manner. This is through the use of healthy gait data which removes variability of gait and compensatory patterns of pathologically affected gait, thus eliminating the need for long-term studies and large subject numbers. If the study used volunteers with gait abnormalities, large subject numbers would be needed to find the typical effect of the PAFO, due to the variability in gait of pathologically affected persons. Furthermore, long-term studies would be required to remove the influence of existing compensatory gait patterns by ensuring subjects had used the PAFO for extended periods of time, such that their gait could adapt and more closely represent restored gait kinematics. Thus, by utilizing healthy gait data and simulating the pathology and PAFO assistance, many variables can be removed, such that the effect of the PAFO mass and mass distribution alone can be clearly identified.

### Limitations

This study utilized healthy gait data as to determine the effects of PAFO mass and mass distribution on kinetics of gait; however, by using the ideal gait kinematics as the assumed gait with the PAFO, the existence of foot drop symptoms and effect of additional mass on gait kinematics have not been included. This is such that it is assumed the PAFO actuator is able to idyllically compensate for the muscles affected by foot drop, such that the exact normal joint angles are achieved. In reality, while it may be possible to adequately compensate for the ankle joint motion, the additional mass added to the lower limb would result in adjusted gait kinematics, such as shorter swing phase and asymmetrical gait [6, 14, 23, 25, 40]. Furthermore, persons with foot drop likely have developed compensatory gait patterns prior to the use of the PAFO and so this may affect their ability to achieve a normal gait, even with an ideal PAFO. The realistic effect of the PAFO mass, with the affected gait kinematics, could only be achieved through long-term clinical studies, while the aim of this simulation study is to understand the effect of the PAFO in a non-destructive and generically predictive manner. Therefore, through these assumptions, it is possible to identify the effect on muscle forces if an ideal gait was to be achieved with the PAFO, and how mass and mass distribution can be optimized to reduce these potential effects.

## Conclusion

This simulation study aimed to determine the effect of PAFO design in terms of mass and mass distribution on net joint moments and individual muscle forces. Through the use of OpenSim, it was possible to rapidly predict the effect of the PAFO on the kinetics of gait, without the need for time-consuming prototyping and testing, or long-term studies. While it was found that higher and laterally mounted actuator positions resulted in less negatively affected net joint moments and overall muscle forces of the lower limb, the effects of the PAFO mass distribution on individual muscle forces must also be considered to avoid detrimental effects caused by either greatly increased or reduced muscle forces. By optimizing the PAFO mass distribution such that gait kinetics can be achieved that are as similar as possible to those of normal gait, it may be possible to reduce the occurrence of potentially detrimental long-term effects and improve rehabilitation outcomes. Therefore, this simulation study could guide future PAFO design in order to improve these devices such that better gait kinematics and kinetics could be achieved, and consequently rehabilitation of the lower limb could be improved. While this study aimed to explore the effect of a PAFO on foot drop affected persons, future research would include further application of these methods to predict the effects on other joints and pathologies, such as the effects of a powered knee and hip orthoses on a hemiparesis-affected person.

## Supplementary Information

Below is the link to the electronic supplementary material.

Supplementary file1 (DOCX 33 KB)
