# Supplementary material for: Effects of powered ankle–foot orthoses mass distribution on lower limb muscle forces—a simulation study
Source: Med Biol Eng Comput. 2023 Jan 23;61(5):1167–82. doi: 10.1007/s11517-023-02778-2 (PMC10083162; doi:10.1007/s11517-023-02778-2)
Supplement: Supplementary file 1 — Supplementary file1 (DOCX 33 KB) [file 11517_2023_2778_MOESM1_ESM.docx]

Supplementary Material

# Online Resource 1

## Muscles included in the OpenSim model

- Adductor longus
- Adductor brevis
- Adductor magnus
- Biceps femoris long head
- Biceps femoris short head
- Erector spinae
- Extensor digitorum longus
- Extensor hallucis longus
- External oblique
- Flexor digitorum longus
- Flexor hallucis longus
- Gemellus
- Gluteus maximus
- Gluteus medius
- Gluteus minimus
- Gracilis
- Iliacus
- Internal oblique
- Lateral gastrocnemius
- Medial gastrocnemius
- Piriformis
- Pectineus
- Psoas
- Peroneus brevis
- Peroneus longus
- Peroneus tertius
- Quadratus femoris
- Rectus femoris
- Sartorius
- Semimembranosus
- Semitendinosus
- Soleus
- Tensor fasciae latae
- Tibialis anterior
- Tibialis posterior
- Vastus intermedius
- Vastus lateralis
- Vastus medialis

# Online Resource 2

## Inverse dynamics and static optimisation equations

Inverse dynamics utilises the equations of motion, shown in equation 1. Where N is the number of model degrees of freedom, $q,\dot{q}, \ddot{q}\in R^{N}$ are the vectors of the generalised positions, velocities, and accelerations and $M\left( q \right)\in R^{N\times N}$ is the system mass matrix. $C(q,\dot{q})\in R^{N}$ is the vector of Coriolis and centrifugal forces, $G(q)\in R^{N}$ is the vector of gravitational forces and $\tau\in R^{N}$ is the vector of generalised forces [36].

$M\left( q \right)\ddot{q}+C\left( q,\dot{q} \right)+G\left( q \right)= \tau$ (1)

Static optimisation utilises equation 2 and equation 3, which depict the objective function for the sum of squared muscle activations and the muscle force-length-velocity function, respectively [36]. Where, $n$ is the number of muscles in the model, $a_{m}$ is the activation level of muscle $m$ at a discrete time point and $F_{m}^{0}$ is the muscles maximum isometric force. $l_{m}$ is the muscle length, $v_{m}$ is its shortening velocity and $\left( F_{m}^{0}, l_{m}, v_{m} \right)$ is the muscles force-length-velocity surface. $r_{m,j}$ is the muscle moment arm about the $j^{th}$ joint axis, and $\tau_{j}$ is the generalised force acting about the $j^{th}$ joint axis [36].

$J= \sum_{m=1}^{n} {(a_{m})}^{2}$ (2)

$\sum_{m=1}^{n} \left[ a_{m}f\left( F_{m}^{0}, l_{m}, v_{m} \right) \right]r_{m,j}= \tau_{j}$ (3)

# Online Resource 3

## Table of results and discussion of the percentage change of peak joint moments for each actuator mass and position compared to the no PAFO situation

| **Percentage change of peak joint moments (%)** | | | | | | | | |
| --- | --- | --- | --- | --- | --- | --- | --- | --- |
|  | **PAFO mass (kg)** | **Actuator position** | | | | | | |
|  |  | **Ankle aligned** | **Lower back** | **Lower side** | **Middle back** | **Middle side** | **Upper back** | **Upper side** |
| **Knee extensor peak moment** | 1 | 113.18 | 105.26 | 106.50 | 102.31 | 103.95 | 102.29 | 103.89 |
|  | 2 | 116.03 | 105.40 | 107.85 | 99.48 | 102.78 | 99.43 | 102.65 |
|  | 3 | 123.71 | 105.78 | 109.40 | 96.87 | 101.81 | 96.80 | 101.62 |
|  | 4 | 133.93 | 108.14 | 110.87 | 94.15 | 100.75 | 94.06 | 100.49 |
|  | 5 | 144.13 | 111.93 | 112.70 | 92.08 | 99.66 | 91.29 | 99.33 |
| **Knee flexor peak moment** | 1 | 421.57 | 387.71 | 384.23 | 375.50 | 370.33 | 367.88 | 363.23 |
|  | 2 | 445.45 | 418.56 | 411.61 | 394.14 | 383.80 | 378.86 | 369.6 |
|  | 3 | 490.88 | 450.55 | 440.12 | 413.91 | 398.41 | 391.00 | 377.11 |
|  | 4 | 535.74 | 481.97 | 468.07 | 433.12 | 412.45 | 402.58 | 384.04 |
|  | 5 | 580.67 | 513.45 | 496.08 | 452.39 | 426.55 | 414.21 | 391.05 |
| **Hip extensor peak moment** | 1 | 323.95 | 282.02 | 280.94 | 265.30 | 263.68 | 252.34 | 251.05 |
|  | 2 | 353.75 | 319.64 | 317.48 | 286.20 | 282.97 | 260.28 | 257.69 |
|  | 3 | 409.87 | 358.76 | 355.45 | 308.59 | 303.69 | 269.67 | 265.78 |
|  | 4 | 465.26 | 397.14 | 392.71 | 330.26 | 323.69 | 278.36 | 273.17 |
|  | 5 | 520.77 | 435.65 | 430.08 | 352.04 | 343.81 | 287.16 | 280.68 |
| **Hip flexor peak moment** | 1 | 88.62 | 76.80 | 77.99 | 72.55 | 74.12 | 70.48 | 71.96 |
|  | 2 | 96.43 | 85.69 | 88.03 | 77.32 | 80.38 | 73.18 | 76.09 |
|  | 3 | 111.09 | 95.16 | 98.61 | 82.73 | 87.26 | 76.54 | 80.84 |
|  | 4 | 125.56 | 104.53 | 109.08 | 88.16 | 94.11 | 79.94 | 85.58 |
|  | 5 | 140.10 | 114.10 | 119.70 | 93.84 | 101.16 | 83.62 | 90.54 |

The knee peak extensor moment occurs at approximately 60% of the gait cycle which aligns with the start of the swing phase at toe off, when the knee continues to bend but is now supporting the shank and foot mass. For the ankle aligned, lower back and lower side positions, the heavier masses cause a greater increase in the extensor moment with the ankle aligned and 5kg mass having the greatest percentage change increase of 144.13%. Interestingly, lower masses cause the greatest effect on knee extensor moment for all middle and upper positions, with 1kg having an increased negative effect as compared to 5kgs. The upper back and 5kg situation had the least effect on knee extensor moment with a percentage change increase of 91.29%. Furthermore, actuators positioned on the side of the limb have a slightly increased effect on knee peak extensor moment.

At 60% of the gait cycle the hip flexor peak moment also occurs, as the hip continues to flex but is now supporting the complete lower limb as swing phase begins. For all positions the heavier masses and lower positions have an increased effect on hip flexor moment as compared to the lighter masses and higher positions. The ankle aligned and 5kg situation results in the greatest effect with a 140.10% increase in percentage change of the hip peak flexor moment, and side mounted positions have as slightly greater effect, as compared to the back mounted positions.

The knee peak flexor moment and the hip peak extensor moment both occur at approximately 90% of the gait cycle, which aligns with terminal swing. At this point in the gait cycle, both the knee and hip reach full extension and begin to slightly flex in preparation for heel strike. In all actuator positions, the knee peak flexor moment and hip peak extensor moment is increased with increasing mass, such that 1kg has the least effect and 5kg has the greatest effect on peak flexor moment. Furthermore, the lower actuator positions are more effected than higher positions and as such the ankle aligned and 5kg situation has the greatest effect, with a percentage change of 580.67% increase on peak knee flexor moment and a 520.77% increase on peak hip extensor moment. For both the knee flexor moment and hip extensor moment, back
